# Seminal exosomes and HIV‐1 transmission

**DOI:** 10.1111/and.13220

**Published:** 2018-12-20

**Authors:** Louise A. Ouattara, Sharon M. Anderson, Gustavo F. Doncel

**Affiliations:** ^1^ CONRAD, Eastern Virginia Medical School Norfolk Virginia

**Keywords:** cell‐to‐cell communication, exosomes, extracellular vesicles, HIV‐1, semen

## Abstract

Exosomes are endosomal‐derived membrane‐confined nanovesicles secreted by many (if not all) cell types and isolated from every human bodily fluid examined up to now including plasma, semen, vaginal secretions and breast milk. Exosomes are thought to represent a new player in cell‐to‐cell communication pathways and immune regulation, and be involved in many physiological and pathological processes. Susceptibility to HIV‐1 infection can be impacted by exosomes, while HIV‐1 pathogenesis can alter exosomal function and composition. Exosomes isolated from semen and vaginal fluid of healthy individuals can inhibit HIV‐1 infection and/or potently block viral transfer in vitro. However, the role of exosomes in HIV‐1 transmission and progression is not fully understood yet and some studies show conflicting results, mainly for exosomes isolated from plasma and breast milk. Determining the composition of exosomes from infected donors and studying their interaction with HIV‐1 in vitro compared to exosomes isolated from uninfected donors will provide insights into the role exosomes play in HIV‐1 transmission during sexual intercourse and breastfeeding.

## 1 INTRODUCTION

Exosomes, small extracellular nanovesicles (30–100 nm) were first discovered few decades ago (Harding, Heuser, & Stahl, 1983; Pan & Johnstone, 1983). Initially considered as a means of discarding cellular waste, exosomes were later found to play an important role in intercellular communications. Exosomes contain a variety of small molecules such as tetraspanins, cytokines, chemokines, lipids, mRNA and microRNA (Raposo & Stoorvogel, 2013; Turchinovich, Samatov, Tonevitsky, & Burwinkel, 2013; Vojtech et al., 2014). Exosomes are encircled by a lipid bilayer, resulting from the inward budding of membrane of the late endosomal compartment. Generated when multivesicular bodies (of late endosomes) fuse with the plasma membrane, exosomes are then released from intraluminal vesicles into the extracellular compartment (Bobrie, Colombo, Raposo, & Thery, 2011; Harding et al., 1983; Simons & Raposo, 2009). Once released in the extracellular compartment, exosomes can break down and release their content into that extracellular compartment or stay intact and interact with target cells by direct fusion with the plasma membrane (Ellwanger, Veit, & Chies, 2017). They deliver their cargo to recipient cells and thereby modulate the host cell function, including innate immune response via the transfer of signalling molecules and antigens (Li et al., 2013; Schorey, Cheng, Singh, & Smith, 2015). Through this mechanism, exosomes represent a new player in cell‐to‐cell communication pathways. In addition to cells, exosomes have been isolated from a variety of different bodily fluids, such as urine (Pisitkun, Shen, & Knepper, 2004), saliva (Palanisamy et al., 2010), blood (Caby, Lankar, Vincendeau‐Scherrer, Raposo, & Bonnerot, 2005), breast milk (Admyre et al., 2007), vaginal fluids (Smith & Daniel, 2016) and semen (Madison, Roller, & Okeoma, 2014; Poliakov, Spilman, Dokland, Amling, & Mobley, 2009; Renneberg, Konrad, Dammshauser, Seitz, & Aumuller, 1997; Sullivan, Saez, Girouard, & Frenette, 2005; Vojtech et al., 2014). Several methods of exosomes isolation have been investigated including serial steps of ultracentrifugation, polymer‐based exosomal precipitation, bead‐based immunologic method and nanotrap particles (Anderson, Kashanchi, & Jacobson, 2018) with each method presenting its advantages and drawbacks.

In this review, we will briefly summarise how exosomes derived from multiple biological matrices compared to seminal exosomes modulate HIV‐1 infection, with a focus on regulation of immune responses.

## 2 EXOSOMES AND HIV‐1: CHARACTERISTICS AND IMPACT

HIV‐1 and exosomes are generally similar in their size (Gentile et al., 1994) and density range (Cimarelli & Luban, 2000), making it difficult to purify exosomes from HIV‐1 infected cells and fluids. Moreover, retroviruses such as HIV‐1 and exosomes share common molecular properties in addition to morphological similarities (Cantin, Diou, Belanger, Tremblay, & Gilbert, 2008). Both exosomes and retroviruses contain major histocompatibility complex class II (MHC‐II) molecules, cell surface molecules such as integrins (CD11a, CD18), co‐stimulatory molecules (CD28, CD54) and complement neutralising molecules (CD55, CD59). However, HIV‐1 particles could be separated from exosomes by using an iodixanol (Optiprep™) velocity gradient as HIV‐1 and exosomes sedimentation velocities are significantly different (Cantin et al., 2008). Exosomes may impact retroviral infection based on biological similarities (Teow, Nordin, Ali, & Khoo, 2016), either through HIV‐1 originating in the same intracellular system as exosomes and using the exosome biogenesis pathway to assemble, disseminate and ultimately infect other cells, or via retroviruses hijacking the mechanism of intercellular communication, including exosomes, to spread and infect other cells. Several additional mechanisms by which exosomes can affect HIV‐1 infection have also been proposed in a more recent review (Ellwanger et al., 2017). HIV‐1 can either release its viral proteins and RNA into exosomes or release virus particles surrounded by exosomes, or viral proteins can interfere with the exosomal release pathway. For example, Nef protein has been shown to reduce the expression of CD4 antigens in exosomes isolated from infected cells, in turn promoting HIV‐1 replication (Arenaccio, Chiozzini, Columba‐Cabezas, Manfredi, Affabris, et al., 2014; de Carvalho et al., 2014). CD4 molecules were present in exosomes secreted from CD4^+^ T cells, and exosomes released by CD4^+^ T cells but not by CD4^−^ T cells were able to efficiently inhibit HIV‐1 infection in vitro. It has been suggested that CD4 molecules displayed on the exosome surface can bind to envelop proteins of HIV‐1, masking the virus interaction with target cells and preventing infection. The authors show that HIV‐1 accessory protein Nef highly expressed in HIV‐1‐infected cells is associated with depletion of CD4 molecules both on cells and released exosomes due to the mechanism, by which Nef promotes targeting of CD4 molecules to multivesicular bodies and then to lysosomes for degradation. Notably, CD4^+^ cells expressing Nef secreted exosomes with weakened inhibitory ability against HIV‐1 infection.

While evidence supports that depending on the cellular source and the protocol of isolation/purification, exosomes can either stimulate or suppress immune responses (Hosseini, Fooladi, Nourani, & Ghanezadeh, 2013; Teow et al., 2016; Thery, Ostrowski, & Segura, 2009; Zhang & Grizzle, 2011) and facilitate or inhibit HIV‐1 infection/transmission (Table 1), the exact role that exosomes play in HIV‐1 infection has not been clearly elucidated. Furthermore, accumulating data suggest that exosomes may impact multiple stages of HIV‐1 pathogenesis by modulating either target immune responses that alter infection, or by activating the latent viral reservoirs (Poveda & Freeman, 2017).

**Table 1:** Summary of exosome effects on HIV‐1 infection in vitro

Exosome source	HIV status	Cell type	Impact on HIV‐1 infection/transmission/disease progression	References
Semen	Negative	Lymphocytes cell lines Monocytic cell lines Primary peripheral blood lymphocytes TZM‐bl cells	Decrease	Madison et al. (2014)
		From vaginal cells to CD4, CCR5 and CXCR4 receptors	Decrease trans‐infection and cell‐to‐cell spreading	Madison et al. (2015)
Vaginal fluid	Negative	293 T/17 cells Jurkat clone E6‐1 T cells	Decrease	Smith and Daniel, (2016)
Breast milk	Negative	Monocyte‐derived dendritic cells (MDDCs)	Decrease	Naslund et al., (2014)
		From MDDCs to CD4^+^ T cells	Decrease viral transfer	
		T lymphoblastoid cell line Macrophage‐like cell line	Increase	Sims et al. (2017, 2018)
Plasma	Negative	Monocyte‐derived dendritic cells (MDDCs)	No significance	Naslund et al., (2014)
		From MDDCs to CD4^+^ T cells	No significance	
	Not specified	T lymphoblastoid cell line Macrophage‐like cell line	Increase	Sims et al. (2017, 2018)
	Positive		Increase	Konadu et al. (2016), Hubert et al. (2015)

## 3 PLASMA EXOSOMES AND HIV‐1 INFECTION

While the mechanisms underlying exosomal regulation of HIV‐1 transmission are not known, it appears that exosomal morphology and function may be altered in HIV‐1 infected individuals. Studies performed with plasma samples have shown that the content of cytokines and chemokines (Konadu et al., 2016), as well as the quantity and size (Hubert et al., 2015), of plasma exosomes from HIV‐1‐infected subjects were significantly higher compared with those of uninfected controls. Interestingly, these changes have shown good correlation with markers of chronic immune activation and disease progression. Both naive and central memory CD4^+^ and CD8^+^ T cells demonstrated a significant increase in expression of activation marker CD38 following exposure to plasma exosomes isolated from HIV‐1‐infected individuals ART‐naive compared to those from seronegative individuals and untreated controls (Hubert et al., 2015). Trans‐activating response (TAR) element RNA, a pre‐miRNA needed for activation of the viral promoter and viral replication through the binding to HIV‐1 Tat protein and interaction with the LTR promoter (Anderson et al., 2018), was detected in the sera from HIV‐1‐positive patients under highly active antiretroviral therapy (HAART), showing that short viral transcripts are still present in exosomes from patients with undetectable viral loads (Jaworski et al., 2014). ART regimens are able to suppress HIV‐1 replication; nevertheless, low CD4 T‐cell counts, immune activation and chronic inflammation can persist and are frequently associated with oxidative stress and elevated rates of comorbidities. Recently, it has been shown that monocytic cells treated with plasma exosomes derived from patients undergoing HAART induced expression of genes related to interferon responses and immune activation (Chettimada et al., 2018). These results suggest that plasma exosomes from patients using antiretrovirals may have pro‐inflammatory effects promoting immune activation and oxidative stress and contributing to the chronic inflammation associated with HIV‐1 disease progression and comorbidities even with suppressed viral loads. Other published data have shown that plasma exosomes enhance HIV‐1 infection in human immune cell lines, with no indication on the patients’ serology (Sims et al., 2017, 2018). In contrast, plasma exosomes isolated from healthy, HIV‐1‐negative donors did not impact HIV‐1 infection of monocyte‐derived dendritic cells or HIV‐1 transfer from those cells to CD4^+^ T cells (Naslund et al., 2014). This suggests that exosomes derived from the same source (plasma) isolated from seronegative and infected donors may have opposing effects on HIV‐1 pathogenesis. In addition, blood‐derived exosomes from HIV‐1 negative donors do not seem to have an impact on HIV‐1 replication and other sexually transmitted virus such as herpes simplex virus type 2 (HSV‐2) (Madison et al., 2014).

HIV‐1 infection induces exosome release from immune cells, including dendritic and T cells (Izquierdo‐Useros et al., 2009; Lenassi et al., 2010; Wiley & Gummuluru, 2006). Exosomes from HIV‐1 infected cells also suppress apoptosis and increase pro‐inflammatory cytokines in naïve target cells, making them more permissive to HIV‐1 infection (Arenaccio, Chiozzini, Columba‐Cabezas, Manfredi, & Federico, 2014; Narayanan et al., 2013; Sampey et al., 2016). It has been shown that exosomal TAR RNA could stimulate pro‐inflammatory cytokines in recipient cells through the nuclear factor kappa b pathway and enhance the susceptibility of naïve cells to HIV‐1 infection. Exosomes isolated from uninfected cells are protective, while exosomes isolated from HIV‐1‐infected lymphocytes and macrophages enhance HIV‐1 infection and replication in target cells (Arenaccio, Chiozzini, Columba‐Cabezas, Manfredi, Affabris, et al., 2014; Kadiu, Narayanasamy, Dash, Zhang, & Gendelman, 2012). However, Barclay et al. (Barclay et al., 2017) showed that exosomes from HIV‐1 uninfected T‐cell lines (CEM, Jurkat) and monocytes (U937) have the possibility to reactivate latent HIV‐1 in T cells (Jurkat E4 cell line, ACH2) and monocyte line (U1), possibly increasing RNA polymerase II activity in the infected cells, facilitating transcription and consequently leading to increased cellular activation. While in vitro mechanistic studies thus far have demonstrated that HIV‐1 infection can both alter exosome composition and function, as well as be impacted by exosomal regulation, the full implications for how plasma and immune cell‐derived exosomes may affect HIV‐1 transmission remain to be determined.

## 4 SEMINAL EXOSOMES AND HIV‐1 INFECTION

### 4.1 Morphology and composition of seminal exosomes

Seminal plasma contains trillions of exosomes (Vojtech et al., 2014). Analyses of semen of 12 different healthy donors revealed more than 10^12^ particles per ejaculate with a range between 4.7 × 10^11^ and 3.12 × 10^13^ SE/ml. Seminal exosome (SE) size is ranging between 50 and 200 nm, a size a little bigger than general exosomes size. Transmission electron microscopy analysis of SE samples has shown that they are composed of lipid bilayer particles in the characteristic size range of exosomes and nanovesicles from other cellular sources. SE have also been shown to express the universal exosome markers heat shock protein (HSP)‐70 and CD63, but not ER calnexin indicating that the vesicles are coming from non‐endoplasmic reticulum sources (Vojtech et al., 2014). These data together with other published data (Madison et al., 2014; Pucci et al., 2017) strongly suggest that the previously described seminal extravesicles called prostasomes identified in human seminal fluid by Poliakov et al. (Poliakov et al., 2009) are indeed exosomes and represent a predominant component of human semen. SE are comprised of proteins, small RNA (miRNA) and mRNA that code for antiviral factors such as host restriction factors (Vojtech et al., 2014). SE contain a high amount of RNA from 20 to 100 nucleotides in length, mainly from extracellular origin. The higher proportion of extracellular RNA in semen is protected within the intact exosomal fraction and is transported to target cells by exosomes (Vojtech et al., 2014). Very little variation among different donors has been observed, indicating high inter‐donor conservation of exosomal composition.

### 4.2 Role in HIV‐1 infection

Semen is known to have both immunostimulatory and immunosuppressive properties, and mediates HIV‐1 transmission through a complex interplay of biological factors in semen and the female genital tract (Doncel, Anderson, & Zalenskaya, 2014; Doncel, Joseph, & Thurman, 2011; Introini et al., 2017; Parsons et al., 2016; Sabatte et al., 2011; Selva, Kent, & Parsons, 2017). On its own, semen exhibits intrinsic antiviral activity that protects against HIV‐1 infection via generation of oxidative products, antimicrobial polypeptides, and the inhibition of HIV‐1 binding to pattern recognition receptors and target cells. However, these inhibitory effects are counterbalanced by facilitative seminal plasma activity that could promote transmission, such as neutralisation of vaginal pH, amyloid fibril formation, complement opsonisation and semen‐mediated pro‐inflammatory responses that culminate in recruitment and activation of immune cells (Doncel et al., 2014, 2011). The potential protective properties of semen were first illustrated by Carlsson et al. and Kitamura et al. showing that prostasomes could inhibit bacterial growth (Carlsson, Pahlson, Bergquist, Ronquist, & Stridsberg, 2000) and measles virus infection (Kitamura et al., 1995).

HIV‐1 transmission occurs mostly through sexual contact, where semen (and SE) from infected individuals come in contact with the genital and colorectal mucosae of uninfected partners. The contribution of SE in semen‐induced mucosal immune responses and impact on HIV‐1 sexual transmission are suspected, but have yet to be properly characterised. One hypothesis is that HIV‐1 infection changes the immunosuppressive function of SE, thus enabling SE from HIV‐1‐infected individuals to promote HIV‐1 sexual transmission and impair the induction of a protective immune response against the infecting virus. This functional shift in SE may be due to hijacking of exosomal pathways by viral proteins, such as HIV‐1 Nef, resulting in a reduced ratio of exosomal versus host cell viral binding sites, which would enhance HIV‐1 entry into target cells (de Carvalho et al., 2014; Kadiu et al., 2012).

Since there is considerable overlap in the cellular generation of enveloped viruses (including HIV‐1) and exosomes (Gould, Booth, & Hildreth, 2003; Madison & Okeoma, 2015), another hypothesis is that HIV‐1 triggers alterations in SE composition and signalling. These phenotypic changes may promote a cascade of functional effects, such as increased cytokine expression and target cell activation, with decreased DC maturation, which collectively compromise the induction of a protective immune response and promote HIV‐1 infection in the female genital mucosa. SE isolated from the semen of healthy donors are generally immunosuppressive, inhibiting the activation of macrophages, NK cells and T cells (Kelly & Critchley, 1997; Kelly et al., 1991; Skibinski, Kelly, Harkiss, & James, 1992; Tarazona et al., 2011; Vojtech et al., 2014). SE may therefore work in tandem with other components of semen, such as prostaglandins, to render the female reproductive tract less responsive to the presence of sperm antigens. This immune response favours conditions for fertilisation but could impact susceptibility to viral infection.

Exosomes from seronegative human semen have been shown to inhibit HIV‐1 cellular entry and transmission in vitro. In TZM‐bl cells, HIV‐1 inhibition was more pronounced when SE were first pre‐incubated with HIV‐1 at 37°C, before exposure to the target cells (Madison et al., 2014), showing a direct effect of SE on the virus. Madison et al. showed that human vaginal epithelial cells V248 internalised SE by endocytosis and direct fusion with the cellular plasma membrane (Madison, Jones, & Okeoma, 2015). Once internalised, SE are able to efficiently prevent transmission of HIV‐1 infection from vaginal epithelial cells to CD4^+^CCR5^+^ monocytic cells and CD4^+^CXCR4^+^ T lymphocytes, as well as peripheral blood mononuclear cells (Madison et al., 2015). In vivo, SE can block intravaginal replication of murine AIDS (mAIDS) virus and limit the disease progression in mice (Madison et al., 2015). SE appear to inhibit HIV‐1 replication post‐virus entrance by disrupting reverse transcriptase (RT) activity, thereby impairing subsequent infectivity (Madison & Okeoma, 2015; Madison et al., 2014). Welch et al. recently showed that SE inhibit the binding of host transcription factors components such as NF‐kB and SP1 to HIV‐1 promoter and the interaction between Tat protein, involved in RT and gene expression, and those components (Welch, Kaddour, Schlievert, Stapleton, & Okeoma, 2018). Although SE attenuate viral RT activity, viral replication in the presence of these exosomes possess the same amount of p24, but less viral RNA (Gag), than the viral progeny generated in the absence of SE. While SE are able to block HIV‐1 and mAIDS virus complex infections, they have no effect on HSV types 1 and 2 replication (Madison et al., 2014), suggesting that the antiviral activity of SE is restricted to HIV‐1 or more generally to retroviruses. However, HSV, perhaps similarly to HIV‐1, seems to hijack exosomes in different compartments modifying their composition and utilising them to disseminate to and infect other cells (Heikkila, Ryodi, & Hukkanen, 2016; Kalamvoki & Deschamps, 2016; Sadeghipour & Mathias, 2017). The properties of SE isolated from HIV‐1‐infected patients have not been fully explored, and this represents a gap in the clear understanding of the role of exosomes in HIV‐1 transmission.

## 5 OTHER REPRODUCTIVE SYSTEM EXOSOMES AND HIV‐1 INFECTION

### 5.1 Vaginal fluid exosomes

Similar to human semen, human vaginal fluid contains exosomes which have been found to reduce HIV‐1 vector transmission by 60% in 293 T/17 cells (Smith & Daniel, 2016). Integrated viral DNA and total viral DNA were both decreased by 47% and 58.4%, respectively, compared to the controls, in the presence of vaginal fluid exosomes (VFE). However, the efficiency of HIV‐1 vector entry into target cells was not significantly different in the presence or absence of these exosomes (Smith & Daniel, 2016). A similar decrease in HIV‐1 transmission was also observed in Jurkat Clone E6‐1 T cells in the presence of VFE by the same authors. The decrease in integration efficiency in presence of VFE could be due to the inhibitory effect of VFE on reverse transcription step. Because the HIV‐1 vector used in those experiments has a different tropism and entry mechanism than HIV‐1 virus, further research is needed to determine exactly the role of VFE in viral cervicovaginal infections by using the virus itself.

### 5.2 Breast milk exosomes

Conflicting reports have been published on exosomes isolated from human breast milk from healthy donors. Indeed, pre‐exposure to exosomes purified from human breast milk inhibits HIV‐1 infection mediated by dendritic cells (DC) by binding to DC‐SIGN, and blocking transport and transfer of virus to target CD4^+^ T cells (Naslund et al., 2014). This might suggest that exosomes from milk may be protective against HIV‐1 transmission from mother to child through breastfeeding. Two more recent papers (Sims et al., 2017, 2018), however, show that exosomes isolated from human breast milk can enhance HIV‐1 entry when exosomes and HIV‐1 are added at the same time to target cells (human CD4^+^ lymphoblastoid T and macrophage‐like cell lines) and co‐incubated for 3 days. These contradictory data may be the result of differences in exosome collection and purification or experimental design. This caveat also applies to many of the discrepant results reported on the functional role of exosomes.

The impact of SE, VFE, plasma, and breast milk exosomes on HIV‐1 infection/transmission is summarised in Table 1.

## 6 RESEARCH PERSPECTIVES

While SE of uninfected individuals have been reported to have the ability to modulate HIV‐1 transmission in vitro, the properties of SE isolated from HIV‐1‐infected patients in acute and chronic stages of the infection have not been fully explored. This represents a critical gap in the knowledge since, globally, HIV‐1 transmission occurs predominantly through sexual transmission. While there is evidence showing that plasma‐derived exosomes from HIV‐1 infected individuals differ in composition and function compared to those from uninfected individuals, the immunomodulatory effect of SE from HIV‐1 seropositive versus seronegative individuals has not been characterised. Given the high amount of SE being delivered to the relatively limited surface area of the CV mucosa and the fact that SE contain abundant cytokines, chemokines, miRNA and mRNA codifying for antiviral factors (Vojtech et al., 2014), it would be important to study how SE exposure modulates immune response and infection in intact CV tissues. Studies aiming to characterise the composition and functional impact of SE obtained from healthy and acutely and chronically infected subjects on immune cells and CV tissues will allow a further understanding of how SE facilitate or impede HIV‐1 acquisition in women. In addition, they will provide insights into the role of SE in HIV‐1 transmission during acute and chronic phases of the infection.

We speculate that exosomes isolated from vaginal fluid and seminal plasma from healthy participants may be used as a model for potential HIV‐1 preventative and therapeutic agents. Delivery systems with exosomal nanoparticles containing anti‐HIV‐1 effectors could be designed and potentially used as therapeutic agents for HIV‐1 prevention and/or treatment. Virus propagation and plasma viraemia were reduced in mice inoculated with a mix of virus and exosomes, suggesting a possible therapeutic function of SE isolated from healthy donors on mucosal and systemic HIV‐1 transmission (Madison et al., 2015; Madison & Okeoma, 2015).

## 7 CONCLUSIONS

Exosomes mediate important cell‐to‐cell communication and, in HIV‐1 infection, this occurs through the exosomal delivery of the viral and host molecules, which vary greatly depending on their cellular source. Exosomes derived from semen of healthy donors can suppress HIV‐1 infection in vitro and decrease intravaginal replication of mAIDS virus. Similarly, exosomes isolated from vaginal fluids can inhibit early steps of HIV‐1 infection. To date, however, the structural and functional properties of SE isolated from HIV‐1 infected subjects have not been reported. Consequently, the exact role of seminal exosomes in HIV‐1 transmission has not been clearly defined, representing a gap in the knowledge and a focus of research with high potential for impact.

## References

[and13220-bib-0001] Admyre, C. , Johansson, S. M. , Qazi, K. R. , Filen, J. J. , Lahesmaa, R. , Norman, M. , … Gabrielsson, S. (2007). Exosomes with immune modulatory features are present in human breast milk. The Journal of Immunology, 179(3), 1969–1978. 10.4049/jimmunol.179.3.1969 17641064

[and13220-bib-0002] Anderson, M. , Kashanchi, F. , & Jacobson, S. (2018). Role of exosomes in human retroviral mediated disorders. Journal of Neuroimmune Pharmacology, 13(3), 279–291. 10.1007/s11481-018-9784-7 29656370

[and13220-bib-0003] Arenaccio, C. , Chiozzini, C. , Columba‐Cabezas, S. , Manfredi, F. , Affabris, E. , Baur, A. , & Federico, M. (2014). Exosomes from human immunodeficiency virus type 1 (HIV‐1)‐infected cells license quiescent CD4^+^ T lymphocytes to replicate HIV‐1 through a Nef‐ and ADAM17‐dependent mechanism. Journal of Virology, 88(19), 11529–11539. 10.1128/jvi.01712-14 25056899 PMC4178784

[and13220-bib-0004] Arenaccio, C. , Chiozzini, C. , Columba‐Cabezas, S. , Manfredi, F. , & Federico, M. (2014). Cell activation and HIV‐1 replication in unstimulated CD4^+^ T lymphocytes ingesting exosomes from cells expressing defective HIV‐1. Retrovirology, 11, 46 10.1186/1742-4690-11-46 24924541 PMC4229896

[and13220-bib-0005] Barclay, R. A. , Schwab, A. , DeMarino, C. , Akpamagbo, Y. , Lepene, B. , Kassaye, S. , … Kashanchi, F. (2017). Exosomes from uninfected cells activate transcription of latent HIV‐1. Journal of Biological Chemistry, 292(28), 11682–11701. 10.1074/jbc.M117.793521 28536264 PMC5512065

[and13220-bib-0006] Bobrie, A. , Colombo, M. , Raposo, G. , & Thery, C. (2011). Exosome secretion: Molecular mechanisms and roles in immune responses. Traffic, 12(12), 1659–1668. 10.1111/j.1600-0854.2011.01225.x 21645191

[and13220-bib-0007] Caby, M. P. , Lankar, D. , Vincendeau‐Scherrer, C. , Raposo, G. , & Bonnerot, C. (2005). Exosomal‐like vesicles are present in human blood plasma. International Immunology, 17(7), 879–887. 10.1093/intimm/dxh267 15908444

[and13220-bib-0008] Cantin, R. , Diou, J. , Belanger, D. , Tremblay, A. M. , & Gilbert, C. (2008). Discrimination between exosomes and HIV‐1: Purification of both vesicles from cell‐free supernatants. Journal of Immunological Methods, 338(1–2), 21–30. 10.1016/j.jim.2008.07.007 18675270

[and13220-bib-0009] Carlsson, L. , Pahlson, C. , Bergquist, M. , Ronquist, G. , & Stridsberg, M. (2000). Antibacterial activity of human prostasomes. Prostate, 44(4), 279–286. 10951492 10.1002/1097-0045(20000901)44:4<279::aid-pros4>3.0.co;2-2

[and13220-bib-0010] Chettimada, S. , Lorenz, D. R. , Misra, V. , Dillon, S. T. , Reeves, R. K. , Manickam, C. , … Gabuzda, D. (2018). Exosome markers associated with immune activation and oxidative stress in HIV patients on antiretroviral therapy. Scientific Reports, 8(1), 7227 10.1038/s41598-018-25515-4 29740045 PMC5940833

[and13220-bib-0011] Cimarelli, A. , & Luban, J. (2000). Human immunodeficiency virus type 1 virion density is not determined by nucleocapsid basic residues. Journal of Virology, 74(15), 6734–6740. 10.1128/JVI.74.15.6734-6740.2000 10888611 PMC112189

[and13220-bib-0012] de Carvalho, J. V. , de Castro, R. O. , da Silva, E. Z. , Silveira, P. P. , da Silva‐Januario, M. E. , Arruda, E. , … daSilva, L. L. (2014). Nef neutralizes the ability of exosomes from CD4^+^ T cells to act as decoys during HIV‐1 infection. PLoS ONE, 9(11), e113691 10.1371/journal.pone.0113691 25423108 PMC4244142

[and13220-bib-0013] Doncel, G. F. , Anderson, S. , & Zalenskaya, I. (2014). Role of semen in modulating the female genital tract microenvironment–implications for HIV transmission. American Journal of Reproductive Immunology, 71(6), 564–574. 10.1111/aji.12231 24702729

[and13220-bib-0014] Doncel, G. F. , Joseph, T. , & Thurman, A. R. (2011). Role of semen in HIV‐1 transmission: Inhibitor or facilitator? American Journal of Reproductive Immunology, 65(3), 292–301. 10.1111/j.1600-0897.2010.00931.x 21087339

[and13220-bib-0015] Ellwanger, J. V. , Veit, T. D. , & Chies, J. A. B. (2017). Exosomes in HIV infection: A review and critical look. Infection, Genetics and Evolution, 53, 146–154. 10.1016/j.meegid.2017.05.021 28546080

[and13220-bib-0016] Gentile, M. , Adrian, T. , Scheidler, A. , Ewald, M. , Dianzani, F. , Pauli, G. , & Gelderblom, H. R. (1994). Determination of the size of HIV using adenovirus type 2 as an internal length marker. Journal of Virological Methods, 48(1), 43–52. 10.1016/0166-0934(94)90087-6 7962259

[and13220-bib-0017] Gould, S. J. , Booth, A. M. , & Hildreth, J. E. (2003). The Trojan exosome hypothesis. Proceedings of the National Academy of Sciences of the United States of America, 100(19), 10592–10597. 10.1073/pnas.1831413100 12947040 PMC196848

[and13220-bib-0018] Harding, C. , Heuser, J. , & Stahl, P. (1983). Receptor‐mediated endocytosis of transferrin and recycling of the transferrin receptor in rat reticulocytes. Journal of Cell Biology, 97(2), 329–339. 10.1083/jcb.97.2.329 6309857 PMC2112509

[and13220-bib-0019] Heikkila, O. , Ryodi, E. , & Hukkanen, V. (2016). The gamma134.5 neurovirulence gene of herpes simplex virus 1 modifies the exosome secretion profile in epithelial cells. Journal of Virology, 90(23), 10981–10984. 10.1128/JVI.01157-16 27630235 PMC5110198

[and13220-bib-0020] Hosseini, H. M. , Fooladi, A. A. , Nourani, M. R. , & Ghanezadeh, F. (2013). The role of exosomes in infectious diseases. Inflammation & Allergy: Drug Targets, 12(1), 29–37. 23441990 10.2174/1871528111312010005

[and13220-bib-0021] Hubert, A. , Subra, C. , Jenabian, M. A. , Tremblay Labrecque, P. F. , Tremblay, C. , Laffont, B. , … Gilbert, C. (2015). Elevated abundance, size, and MicroRNA content of plasma extracellular vesicles in viremic HIV‐1+ patients: Correlations with known markers of disease progression. Journal of Acquired Immune Deficiency Syndromes, 70(3), 219–227. 10.1097/QAI.0000000000000756 26181817 PMC4627170

[and13220-bib-0022] Introini, A. , Bostrom, S. , Bradley, F. , Gibbs, A. , Glaessgen, A. , Tjernlund, A. , & Broliden, K. (2017). Seminal plasma induces inflammation and enhances HIV‐1 replication in human cervical tissue explants. PLoS Path, 13(5), e1006402 10.1371/journal.ppat.1006402 PMC5453613 28542587

[and13220-bib-0023] Izquierdo‐Useros, N. , Naranjo‐Gomez, M. , Archer, J. , Hatch, S. C. , Erkizia, I. , Blanco, J. , … Martinez‐Picado, J. (2009). Capture and transfer of HIV‐1 particles by mature dendritic cells converges with the exosome‐dissemination pathway. Blood, 113(12), 2732–2741. 10.1182/blood-2008-05-158642 18945959 PMC2661860

[and13220-bib-0024] Jaworski, E. , Saifuddin, M. , Sampey, G. , Shafagati, N. , Van Duyne, R. , Iordanskiy, S. , … Kashanchi, F. (2014). The use of Nanotrap particles technology in capturing HIV‐1 virions and viral proteins from infected cells. PLoS ONE, 9(5), e96778 10.1371/journal.pone.0096778 24820173 PMC4018389

[and13220-bib-0025] Kadiu, I. , Narayanasamy, P. , Dash, P. K. , Zhang, W. , & Gendelman, H. E. (2012). Biochemical and biologic characterization of exosomes and microvesicles as facilitators of HIV‐1 infection in macrophages. The Journal of Immunology, 189(2), 744–754. 10.4049/jimmunol.1102244 22711894 PMC3786185

[and13220-bib-0026] Kalamvoki, M. , & Deschamps, T. (2016). Extracellular vesicles during Herpes Simplex Virus type 1 infection: An inquire. Virology Journal, 13, 63 10.1186/s12985-016-0518-2 27048572 PMC4822280

[and13220-bib-0027] Kelly, R. W. , & Critchley, H. O. (1997). Immunomodulation by human seminal plasma: A benefit for spermatozoon and pathogen? Human Reproduction, 12(10), 2200–2207. 10.1093/oxfordjournals.humrep.a019559 9402282

[and13220-bib-0028] Kelly, R. W. , Holland, P. , Skibinski, G. , Harrison, C. , McMillan, L. , Hargreave, T. , & James, K. (1991). Extracellular organelles (prostasomes) are immunosuppressive components of human semen. Clinical and Experimental Immunology, 86(3), 550–556. 10.1111/j.1365-2249.1991.tb02968.x 1747961 PMC1554200

[and13220-bib-0029] Kitamura, M. , Namiki, M. , Matsumiya, K. , Tanaka, K. , Matsumoto, M. , Hara, T. , … Seya, T. (1995). Membrane cofactor protein (CD46) in seminal plasma is a prostasome‐bound form with complement regulatory activity and measles virus neutralizing activity. Immunology, 84(4), 626–632. 7790037 PMC1415160

[and13220-bib-0030] Konadu, K. A. , Huang, M. B. , Roth, W. , Armstrong, W. , Powell, M. , Villinger, F. , & Bond, V. (2016). Isolation of exosomes from the plasma of HIV‐1 positive individuals. Journal of Visualized Experiments, 107 10.3791/53495 PMC4780996 26780239

[and13220-bib-0031] Lenassi, M. , Cagney, G. , Liao, M. , Vaupotic, T. , Bartholomeeusen, K. , Cheng, Y. , … Peterlin, B. M. (2010). HIV Nef is secreted in exosomes and triggers apoptosis in bystander CD4^+^ T cells. Traffic, 11(1), 110–122. 10.1111/j.1600-0854.2009.01006.x 19912576 PMC2796297

[and13220-bib-0032] Li, J. , Liu, K. , Liu, Y. , Xu, Y. , Zhang, F. , Yang, H. , … Yuan, Z. (2013). Exosomes mediate the cell‐to‐cell transmission of IFN‐alpha‐induced antiviral activity. Nature Immunology, 14(8), 793–803. 10.1038/ni.2647 23832071

[and13220-bib-0033] Madison, M. N. , Jones, P. H. , & Okeoma, C. M. (2015). Exosomes in human semen restrict HIV‐1 transmission by vaginal cells and block intravaginal replication of LP‐BM5 murine AIDS virus complex. Virology, 482, 189–201. 10.1016/j.virol.2015.03.040 25880110 PMC4461544

[and13220-bib-0034] Madison, M. N. , & Okeoma, C. M. (2015). Exosomes: Implications in HIV‐1 Pathogenesis. Viruses, 7(7), 4093–4118. 10.3390/v7072810 26205405 PMC4517139

[and13220-bib-0035] Madison, M. N. , Roller, R. J. , & Okeoma, C. M. (2014). Human semen contains exosomes with potent anti‐HIV‐1 activity. Retrovirology, 11, 102 10.1186/s12977-014-0102-z 25407601 PMC4245725

[and13220-bib-0036] Narayanan, A. , Iordanskiy, S. , Das, R. , Van Duyne, R. , Santos, S. , Jaworski, E. , … Kashanchi, F. (2013). Exosomes derived from HIV‐1‐infected cells contain trans‐activation response element RNA. Journal of Biological Chemistry, 288(27), 20014–20033. 10.1074/jbc.M112.438895 23661700 PMC3707700

[and13220-bib-0037] Naslund, T. I. , Paquin‐Proulx, D. , Paredes, P. T. , Vallhov, H. , Sandberg, J. K. , & Gabrielsson, S. (2014). Exosomes from breast milk inhibit HIV‐1 infection of dendritic cells and subsequent viral transfer to CD4^+^ T cells. AIDS, 28(2), 171–180. 10.1097/QAD.0000000000000159 24413309

[and13220-bib-0038] Palanisamy, V. , Sharma, S. , Deshpande, A. , Zhou, H. , Gimzewski, J. , & Wong, D. T. (2010). Nanostructural and transcriptomic analyses of human saliva derived exosomes. PLoS ONE, 5(1), e8577 10.1371/journal.pone.0008577 20052414 PMC2797607

[and13220-bib-0039] Pan, B. T. , & Johnstone, R. M. (1983). Fate of the transferrin receptor during maturation of sheep reticulocytes in vitro: Selective externalization of the receptor. Cell, 33(3), 967–978. 10.1016/0092-8674(83)90040-5 6307529

[and13220-bib-0040] Parsons, M. S. , Madhavi, V. , Ana‐Sosa‐Batiz, F. , Center, R. J. , Wilson, K. M. , Bunupuradah, T. , … Kent, S. J. (2016). Brief report: Seminal plasma anti‐HIV antibodies trigger antibody‐dependent cellular cytotoxicity: Implications for HIV transmission. Journal of Acquired Immune Deficiency Syndromes, 71(1), 17–23. 10.1097/QAI.0000000000000804 26761269

[and13220-bib-0041] Pisitkun, T. , Shen, R. F. , & Knepper, M. A. (2004). Identification and proteomic profiling of exosomes in human urine. Proceedings of the National Academy of Sciences of the United States of America, 101(36), 13368–13373. 10.1073/pnas.0403453101 15326289 PMC516573

[and13220-bib-0042] Poliakov, A. , Spilman, M. , Dokland, T. , Amling, C. L. , & Mobley, J. A. (2009). Structural heterogeneity and protein composition of exosome‐like vesicles (prostasomes) in human semen. Prostate, 69(2), 159–167. 10.1002/pros.20860 18819103

[and13220-bib-0043] Poveda, E. , & Freeman, M. L. (2017). Hot news: Exosomes as new players in HIV pathogenesis – New data from the IAS 2017. AIDS Rev, 19(3), 173–175. 10.24875/AIDSRev.M17000007 29019351 PMC5828156

[and13220-bib-0044] Pucci, M. , Taverna, S. , Reclusa, P. , Pinto, J. A. , Durendez, E. , Lewintre, E. J. , … Rolfo, C. (2017). Exosomes in semen: Opportunities as a new tool in prostate cancer diagnosis. Translational Cancer Research, 6(S8), S1331–S1338. 10.21037/tcr.2017.10.25

[and13220-bib-0045] Raposo, G. , & Stoorvogel, W. (2013). Extracellular vesicles: Exosomes, microvesicles, and friends. Journal of Cell Biology, 200(4), 373–383. 10.1083/jcb.201211138. 23420871 PMC3575529

[and13220-bib-0046] Renneberg, H. , Konrad, L. , Dammshauser, I. , Seitz, J. , & Aumuller, G. (1997). Immunohistochemistry of prostasomes from human semen. Prostate, 30(2), 98–106. 9051148 10.1002/(sici)1097-0045(19970201)30:2<98::aid-pros5>3.0.co;2-g

[and13220-bib-0047] Sabatte, J. , Remes Lenicov, F. , Cabrini, M. , Rodriguez Rodrigues, C. , Ostrowski, M. , Ceballos, A. , … Geffner, J. (2011). The role of semen in sexual transmission of HIV: Beyond a carrier for virus particles. Microbes and Infection, 13(12–13), 977–982. 10.1016/j.micinf.2011.06.005 21767659

[and13220-bib-0048] Sadeghipour, S. , & Mathias, R. A. (2017). Herpesviruses hijack host exosomes for viral pathogenesis. Seminars in Cell & Developmental Biology, 67, 91–100. 10.1016/j.semcdb.2017.03.005 28456604

[and13220-bib-0049] Sampey, G. C. , Saifuddin, M. , Schwab, A. , Barclay, R. , Punya, S. , Chung, M. C. , … Kashanchi, F. (2016). Exosomes from HIV‐1‐infected cells stimulate production of pro‐inflammatory cytokines through trans‐activating response (TAR) RNA. Journal of Biological Chemistry, 291(3), 1251–1266. 10.1074/jbc.M115.662171 26553869 PMC4714213

[and13220-bib-0050] Schorey, J. S. , Cheng, Y. , Singh, P. P. , & Smith, V. L. (2015). Exosomes and other extracellular vesicles in host‐pathogen interactions. EMBO Reports, 16(1), 24–43. 10.15252/embr.201439363 25488940 PMC4304727

[and13220-bib-0051] Selva, K. J. , Kent, S. J. , & Parsons, M. S. (2017). Modulation of innate and adaptive cellular immunity relevant to HIV‐1 vaccine design by seminal plasma. AIDS, 31(3), 333–342. 10.1097/QAD.0000000000001319 27835615

[and13220-bib-0052] Simons, M. , & Raposo, G. (2009). Exosomes–vesicular carriers for intercellular communication. Current Opinion in Cell Biology, 21(4), 575–581. 10.1016/j.ceb.2009.03.007 19442504

[and13220-bib-0053] Sims, B. , Farrow, A. L. , Williams, S. D. , Bansal, A. , Krendelchtchikov, A. , Gu, L. , & Matthews, Q. L. (2017). Role of TIM‐4 in exosome‐dependent entry of HIV‐1 into human immune cells. Int J Nanomedicine, 12, 4823–4833. 10.2147/IJN.S132762 28740388 PMC5505621

[and13220-bib-0054] Sims, B. , Farrow, A. L. , Williams, S. D. , Bansal, A. , Krendelchtchikov, A. , & Matthews, Q. L. (2018). Tetraspanin blockage reduces exosome‐mediated HIV‐1 entry. Archives of Virology, 10.1007/s00705-018-3737-6 PMC5958159 29429034

[and13220-bib-0055] Skibinski, G. , Kelly, R. W. , Harkiss, D. , & James, K. (1992). Immunosuppression by human seminal plasma–extracellular organelles (prostasomes) modulate activity of phagocytic cells. American Journal of Reproductive Immunology, 28(2), 97–103. 1337434 10.1111/j.1600-0897.1992.tb00767.x

[and13220-bib-0056] Smith, J. A. , & Daniel, R. (2016). Human vaginal fluid contains exosomes that have an inhibitory effect on an early step of the HIV‐1 life cycle. AIDS, 30(17), 2611–2616. 10.1097/QAD.0000000000001236 27536982 PMC6141010

[and13220-bib-0057] Sullivan, R. , Saez, F. , Girouard, J. , & Frenette, G. (2005). Role of exosomes in sperm maturation during the transit along the male reproductive tract. Blood Cells, Molecules, and Diseases, 35(1), 1–10. 10.1016/j.bcmd.2005.03.005 15893944

[and13220-bib-0058] Tarazona, R. , Delgado, E. , Guarnizo, M. C. , Roncero, R. G. , Morgado, S. , Sanchez‐Correa, B. , … Casado, J. G. (2011). Human prostasomes express CD48 and interfere with NK cell function. Immunobiology, 216(1–2), 41–46. 10.1016/j.imbio.2010.03.002 20382443

[and13220-bib-0059] Teow, S. Y. , Nordin, A. C. , Ali, S. A. , & Khoo, A. S. (2016). Exosomes in human immunodeficiency virus type I pathogenesis: Threat or opportunity? Archives of Virology, 2016, 9852494 10.1155/2016/9852494 PMC4766318 26981123

[and13220-bib-0060] Thery, C. , Ostrowski, M. , & Segura, E. (2009). Membrane vesicles as conveyors of immune responses. Nature Reviews Immunology, 9(8), 581–593. 10.1038/nri2567 19498381

[and13220-bib-0061] Turchinovich, A. , Samatov, T. R. , Tonevitsky, A. G. , & Burwinkel, B. (2013). Circulating miRNAs: Cell‐cell communication function? Front Genet, 4, 119 10.3389/fgene.2013.00119 23825476 PMC3695387

[and13220-bib-0062] Vojtech, L. , Woo, S. , Hughes, S. , Levy, C. , Ballweber, L. , Sauteraud, R. P. , … Hladik, F. (2014). Exosomes in human semen carry a distinctive repertoire of small non‐coding RNAs with potential regulatory functions. Nucleic Acids Research, 42(11), 7290–7304. 10.1093/nar/gku347 24838567 PMC4066774

[and13220-bib-0063] Welch, J. L. , Kaddour, H. , Schlievert, P. M. , Stapleton, J. T. , & Okeoma, C. M. (2018). Semen exosomes promote transcriptional silencing of HIV‐1 by disrupting NF‐kB/Sp1/Tat circuitry. Journal of Virology, 10.1128/JVI.00731-18 PMC6189507 30111566

[and13220-bib-0064] Wiley, R. D. , & Gummuluru, S. (2006). Immature dendritic cell‐derived exosomes can mediate HIV‐1 trans infection. Proceedings of the National Academy of Sciences of the United States of America, 103(3), 738–743. 10.1073/pnas.0507995103 16407131 PMC1334656

[and13220-bib-0065] Zhang, H. G. , & Grizzle, W. E. (2011). Exosomes and cancer: A newly described pathway of immune suppression. Clinical Cancer Research, 17(5), 959–964. 10.1158/1078-0432.ccr-10-1489 21224375 PMC3155407

